# Biocatalytic Reduction Reactions from a Chemist's Perspective

**DOI:** 10.1002/anie.202001876

**Published:** 2020-11-03

**Authors:** Frank Hollmann, Diederik J. Opperman, Caroline E. Paul

**Affiliations:** ^1^ Department of Biotechnology Delft University of Technology Van der Maasweg 9 2629 HZ Delft The Netherlands; ^2^ Department of Biotechnology University of the Free State 205 Nelson Mandela Drive Bloemfontein 9300 South Africa

**Keywords:** bioreductions, dehydrogenases, hydrogenation, reductases, reductive amination

## Abstract

Reductions play a key role in organic synthesis, producing chiral products with new functionalities. Enzymes can catalyse such reactions with exquisite stereo‐, regio‐ and chemoselectivity, leading the way to alternative shorter classical synthetic routes towards not only high‐added‐value compounds but also bulk chemicals. In this review we describe the synthetic state‐of‐the‐art and potential of enzymes that catalyse reductions, ranging from carbonyl, enone and aromatic reductions to reductive aminations.

## Graphical abstract

Biocatalytic reductions: Enzymes catalyse reduction reactions with exquisite stereo‐, regio‐ and chemoselectivity, leading the way to shorter alternatives to classical synthetic routes!

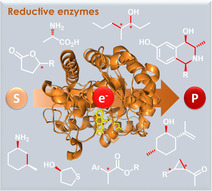

## 1 Introduction

Nature's catalysts, enzymes, provide almost infinite possibilities to access a plethora of chemical reactions. Reductions in particular can lead to the generation of not only multiple chiral centres, but also new functional groups in products highly sought after in the pharma and fine chemical industries. Nowadays, biocatalytic reductions can access compounds such as chiral α‐ or β‐substituted carbonyls, alcohols, secondary amines and lactones (from keto esters).

In this review, we give a critical analysis of the use of oxidoreductases for chemical synthesis. We also provide a perspective on the enzymes currently available to catalyse reductions divided according to the type of chemical reaction. Highlights of preparative‐scale reactions are included to demonstrate the synthetic potential of reductive enzymes in chemical synthesis.

### 1.1 General Considerations

#### 1.1.1 Why Use Enzymes as Reduction Catalysts?

**Figure 1:**
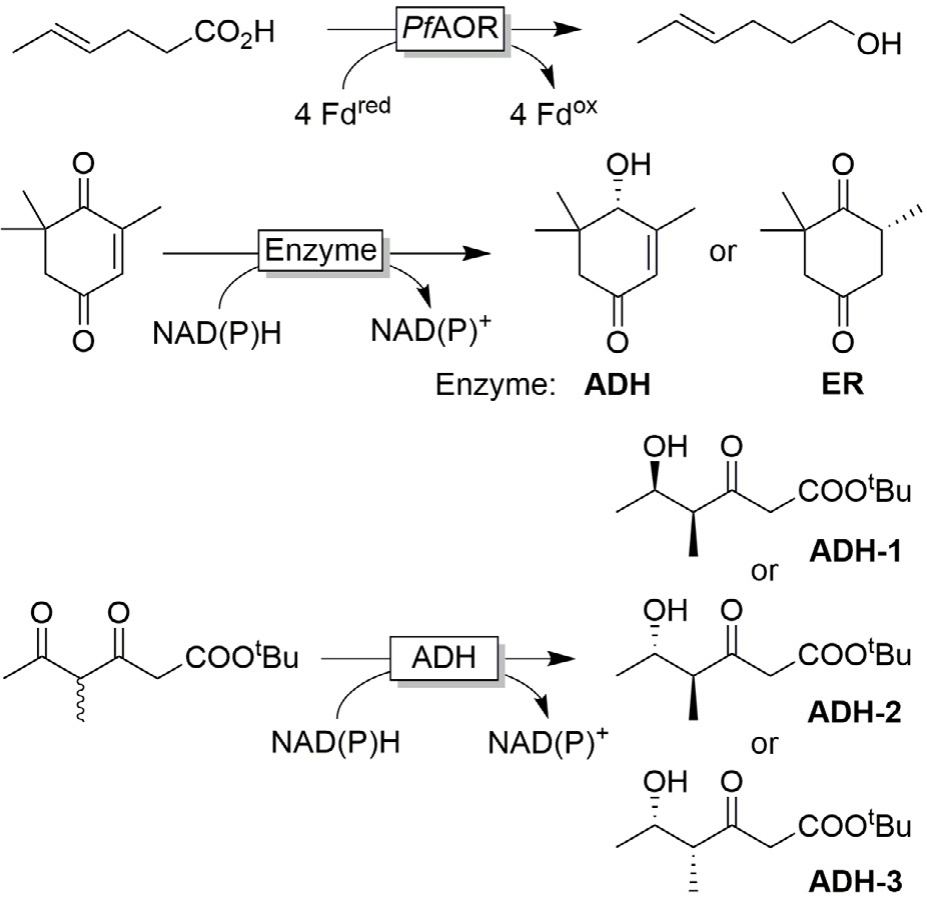
Examples of enzyme selectivity. Chemoselective reduction of carboxylate groups in the presence of other functional groups;^[2]^ chemoselective reduction of C=C or C=O bonds depending on the enzyme used;^[3]^ regio‐ and stereoselective reduction of carbonyl groups with in situ racemisation of an α‐methyl group using different ADH.^[4]^

*Selectivity*! Oxidoreductases, that is, enzymes catalysing oxidation and reduction reactions, are structurally well‐defined polypeptides that place the reducing agent in a distinct, chiral environment enabling them to discriminate between enantiotopic sides of prochiral stereocenters or between different functional groups.^[1]^ Hence, oxidoreductases can control the locus of reduction independent from the chemical reactivity of multiple functional groups present within the starting material. Some examples for selective reductions are the chemoselective reduction of carboxylate groups in the presence of C=C double bonds or carbonyl groups,^[2]^ the chemoselective reduction of either the carbonyl or C=C group in ketoisophorone,^[3]^ and the regio‐ and stereoselective reduction of polyketones^[4]^ (Figure 1). Further examples highlighting several aspects of selectivity will appear throughout this contribution. Finally, consumers are increasingly showing interest in “natural products” in contrast to “nature‐identical products”. Biocatalysis plays a central role in the production of “natural” flavours and fragrances.^[5]^

#### 1.1.2 Are Biocatalytic Reactions Green?

*Possibly*. Today, the statement “biocatalysis is intrinsically green” has become a mantra for many researchers. Arguments such as the mild reaction conditions, the renewable and biodegradable character of the catalysts and the generally aqueous reaction conditions are brought forward to substantiate the “greenness” of biocatalytic reactions. These arguments, however, are rather superficial and generally lack any quantitative substantiation.

First of all, researchers should be aware that no chemical transformation (including biocatalytic reactions) is *green*, as in all cases resources are consumed and waste is generated, thereby putting a burden on the environment. We believe that a given reaction of methodology can be *greener than* another reaction. Such a comparison, however, should be based on quantitative data rather than on general statements. Comparative full life cycle assessments (LCA) represent the “gold standard” for such comparisons, but are usually time‐intensive due to the large data basis required for a meaningful comparison. Sheldon's E‐factor^[6]^ and possibly its derivative, the E^+^‐factor, taking energy‐related CO_2_ emissions into account,^[7]^ represent an acceptable alternative for the preparative chemist.

The mild reaction conditions (particularly the generally low temperature) certainly represent an advantage of biocatalysis over other catalytic methods, as these often also imply energy savings. Furthermore, biocatalytic reactions excel in their higher selectivity, resulting in less tedious downstream processing and product purification. The latter often contribute significantly to the overall resource‐consumption and waste generation.^[8]^ Hence, selective reactions are in general greener than non‐selective reactions.

**Figure 2:**
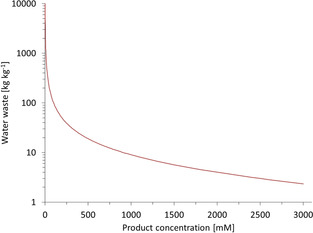
Wastewater incurring at different product concentrations. A molar mass of 100 g mol^−1^ for the product was taken as basis for the calculation.

Water is commonly considered to be a green solvent, which is certainly true prior to the reaction. After almost any (bio)chemical reaction, however, the solvent (water) contains reagents, which have to be removed before the purified water is released into the environment. The high specific heat capacity of water also requires significant energy input for temperature control (and distillative purification). Most importantly, water is a suboptimal solvent for hydrophobic reagents commonly used in biocatalytic reactions. Consequently, rather dilute (1–10 mm) reagent concentrations are still common in the biocatalysis literature. This is unsustainable both from an environmental (Figure 2) and from an economic point of view (vide infra).

Overall, biocatalysis holds an enormous potential for environmentally less demanding synthesis. But biocatalysis is not *intrinsically green* and any green claim needs to be substantiated by at least a semiquantitative comparison with an alternative synthesis route!

#### 1.1.3 Are Biocatalytic Reductions Economically Feasible?

*Yes*. To assess the economic feasibility, many factors have to be taken into account. Amongst them the cost contribution of the (bio)catalyst to the product as well as the reaction costs are discussed briefly.

To assess the cost contribution of the biocatalyst to a final product, the production costs and the performance of the catalyst need to be taken into account. Tufvesson and Woodley have pointed out the dependency of the production costs of whole cells and enzymes on the scale of the fermentation.^[9]^ The production costs for industrial (non‐purified) enzymes range between 250 and 1000 € kg^−1^, whereas the whole cells are considerably cheaper (35–100 € kg^−1^).

**Figure 3:**
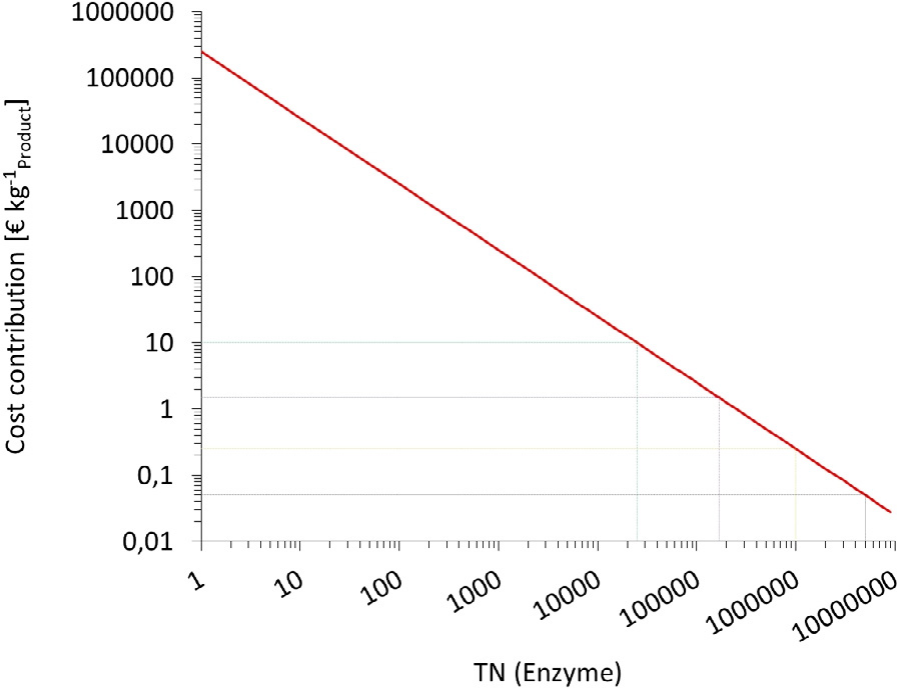
Estimation of the cost contribution of an enzyme to the final product. TN=mol_Product_×mol_Enzyme_^−1^; molecular weights of 100 g mol^−1^ for the product and 50×10^3^ g mol^−1^ for the enzyme and an average production price of 500 € kg^−1^_Enzyme_ for the enzyme were assumed. Dotted lines give the acceptable cost contribution for different products: green: 10 € kg^−1^ (pharma); purple: 1.5 € kg^−1^ (fine chemicals); orange: 0.25 € kg^−1^ (specialty chemicals) and black: 0.05 € kg^−1^ (bulk chemicals).

With these numbers, the cost contribution of an enzyme to the production cost of the product can be easily estimated (Figure 3). As a rule of thumb, to achieve economic feasibility, an enzyme should perform at least 25 000 catalytic cycles for the production of pharmaceuticals and more than 5 000 000 catalytic cycles for the production of bulk chemicals, respectively. Similar (but lower) numbers can also be calculated for whole‐cell biotransformations.[^9^, ^10^]

Next to the biocatalyst of course other factors contribute to the production costs, such as space–time yield and the effort needed for product isolation. In this respect, especially the aforementioned dilute reaction mixtures are unattractive from an economical point of view, as the existing infrastructure is used inefficiently. To address this issue, various concepts to increase the overall product concentration have been developed in the past years. In a two liquid‐phase system approach (2LPS) a hydrophobic organic phase (ideally the starting material itself) serves as the substrate reservoir and the product sink, thereby enabling overall high reagent concentrations. In addition to increasing the reagent concentration, 2LPSs have been used to shift unfavourable reaction equilibria^[11]^ and prevent undesired follow‐up or side reactions.[^2b^, ^12^] Multiphase reactions are not necessarily limited to the 2LPS concept. Slurry‐to‐slurry reactions have been reported, in which a solid, poorly water‐soluble starting material is turned into an insoluble product.^[13]^ Spiess and co‐workers also reported biocatalytic reduction reactions based on gaseous reagents.^[14]^

**Figure 4:**
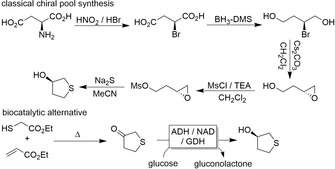
Example of the simplified process for the synthesis of (*R*)‐tetrahydrothiophene‐3‐ol.

The high selectivity of enzymatic transformations can lead to significant process simplifications as exemplified by Codexis for the synthesis of (*R*)‐tetrahydrothiophene‐3‐ol (Figure 4).^[15]^ Non‐enzymatic reduction methods fail in the enantiodiscrimination of the pro‐*R* and pro‐*S* face of tetrahydrothiophene‐3‐one, which is why a five‐step chiral pool synthesis starting from aspartate was used initially (Figure 4 top). An engineered ADH with exclusive enantioselectivity and robustness under process conditions made this tedious sequence obsolete by a single biocatalytic reduction step. In addition, the milder reaction conditions of biocatalytic reactions lead to fewer (e.g. thermal) by‐products, simplifying downstream processing and improving economic and environmental benefits.^[16]^

### 1.2 The Catalysts

Traditionally, carbonyl reductions dominate the field of biocatalytic reduction chemistry; for decades the stereoselective reduction of prochiral ketones has been the dominating transformation.^[17]^ Since the early 2000s, this situation has changed dramatically with the (re‐)discovery of ene reductases for the reduction of conjugated C=C bonds,^[18]^ the reductive amination of ketones and aldehydes by transaminases,^[19]^ amine dehydrogenases,^[20]^ and imine reductases,^[21]^ and more recently the reduction of carboxylic acids by carboxylic acid reductases^[22]^ and aldehyde oxidoreductases.^[23]^

Alcohol dehydrogenases (ADHs) are the catalysts of choice for the reduction of aldehydes and ketones to the corresponding alcohols. ADHs utilise the reduced nicotinamide adenine dinucleotide cofactor (NAD(P)H) as a stoichiometric reductant, transferring a hydride in a reversible manner to the carbonyl C‐atom (Figures S1 and S3 in the Supporting Information).^[1d]^

Similar to ADHs, carboxylic acid reductases (CARs, Figure S9), amine dehydrogenases (AmDHs, Figure S7) and imine reductases (IREDs, Figure S5) also utilise NAD(P)H as reductant in their catalytic mechanisms.

Flavin‐dependent ene reductases (ERs) are worth a brief introduction. Mechanistically, NAD(P)H gives a hydride to the ER′s prosthetic flavin cofactor (Figure S4), which then transfers the hydride in a Michael‐type addition to the bound substrate (containing a conjugated C=C double bond). The catalytic cycle is finalised by protonation, usually in the *trans* configuration (Figure S4).

Finally, the reductive amination of carbonyl groups catalysed by transaminases (TAs) follows an entirely different mechanism (Figure S6). Here, the amine donor itself also serves as the reductant; in the first step the amine donor (such as isopropyl amine or alanine, amongst others) is oxidised to the corresponding ketone, accompanied by a reductive amination of the enzyme‐bound pyridoxal 5′‐phosphate (PLP) cofactor. The amine form of the latter then performs the stereoselective reductive amination of the TA substrate.[^19a^, ^19b^, ^19c^, ^19d^]

In the early days of biocatalysis, only naturally occurring enzymes were available (ADH from horse liver is one example), which obviously severely limited the scope of transformations and products. Today, using enzymes directly from a naturally occurring source has become the exception and recombinant expression of enzymes in heterologous host microbes such as *Escherichia coli* is the norm. Recombinant expression has also paved the way for protein engineering to tailor the properties of a given enzyme to suit the needs of the organic chemist.^[24]^ In essence, any property of a given enzyme ranging from the inhibitory effects of reagents, catalytic activity, stability, substrate range or selectivity can be addressed.[^24^, ^25^]

### 1.3 Sources of Reducing Equivalents

Like any non‐enzymatic reduction reaction, biocatalytic reductions require reducing agents to drive the reaction. For most oxidoreductases, the reduced nicotinamide adenine dinucleotide cofactors (NADH or NADPH, Figure S1) serve as reductants. In the course of the reduction reaction, NAD(P)H is oxidised to NAD(P)^+^. For economic reasons and to avoid possible inhibitory effects of the accumulating oxidised cofactor, its reduced form must be regenerated to enter a new catalytic cycle. For decades, this issue has inspired researchers to search for new regeneration methods.^[26]^ A broad range of NAD(P)H regeneration systems have been developed (Table 1).

**Table 1:** Selection of in situ NAD(P)H regeneration systems. 
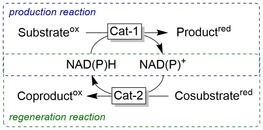

Cosubstrate	Cat‐2	Coproduct (g mol^−1^)	Ref.
H_2_	Hase	– (0)	[32]
HCO_2_H	FDH	CO_2_ (44)	
glucose	GDH	gluconic acid (196)	[15]
isopropanol	ADH	acetone (58)	[13, 33]
MeOH	ADH, AldDH, FDH	CO_2_ (15)	[34]
diols	lactones	variable, but irreversible regeneration reaction	[27c, 28a, 35]
H_2_O	algae	1/2 O_2_ (16)	[36]

For the reduction of carbonyl groups, the coupled‐substrate regeneration approach using isopropanol as a stoichiometric reductant is widely used. Exploiting the reversibility of the ADH reaction, the production enzyme is also used for the regeneration of the nicotinamide cofactor (Table 1, Cat‐1=Cat‐2). This approach saves an additional enzyme and opens up the possibility of using ADHs in non‐aqueous reaction (neat) media because the (exclusively water‐soluble) nicotinamide cofactor does not have to leave the enzyme active site.^[27]^ On the downside, the overall thermodynamic driving force of this approach is rather low, necessitating high molar excesses to shift the equilibrium favourably. To circumvent this, Kara et al. have developed so‐called “smart‐cosubstrate” approaches based on the oxidative lactonisation of diols to the corresponding lactones. This approach is not only irreversible but also doubles the NAD(P)H yield per cosubstrate equivalent (double oxidation of the cosubstrate).^[28]^

Amongst the coupled‐enzyme regeneration approaches, formate and glucose are the cosubstrates when formate dehydrogenase (FDH) or glucose dehydrogenase (GDH) are used as regeneration enzymes, respectively. Both regeneration systems are irreversible but differ significantly with respect to the by‐product. While the FDH reaction yields volatile CO_2_ as a by‐product, which does not accumulate in the reaction mixture,^[29]^ GDH eventually yields gluconic acid, which remains in the reaction medium and lowers the pH significantly.^[30]^ Nevertheless, GDH, due to its high activity and robustness, remains a popular regeneration system.[^10^, ^15^, ^31^]

Hydrogen represents an attractive stoichiometric reductant, as in principle it yields no by‐products.[^32^, ^37^] Current robustness issues of the hydrogenases available, however, call for further improvements.

The cathode represents an equally clean source of reducing equivalents. In the 1990s Steckhan pioneered the indirect electrochemical regeneration of reduced nicotinamide cofactors.^[38]^ After two decades of relative silence around this approach, this topic is now gaining renewed interest as “microbial electrosynthesis”.^[39]^ Essentially, whole cells are more or less directly connected to the cathode as a reagent‐free reductant. Right now, it is too early to tell if this promising approach will be practical.

Finally, the rapidly developing field of algae biotechnology should be mentioned, as algae, enabled by visible light, use water as a stoichiometric reductant to drive reductive cellular processes. Algae can also be used for selective reduction reactions.[^36^, ^40^]

## 2 Reduction of C−C Multiple Bonds

### 2.1 C=C Double Bonds

Especially thanks to the efforts by Faber and Hauer, the stereoselective reduction of conjugated C=C double bonds has now moved into focus of academic and industrial interest.^[41]^ Pioneering works by Simon and co‐workers using whole *Clostridia* cells^[42]^ are less considered nowadays mostly due to the high oxygen sensitivity of these biocatalysts.

Over the past few decades, oxygen‐insensitive ene reductases (ERs) from the Old Yellow Enzyme (OYE) family (E.C. 1.6.99.1) have dominated the field of biocatalytic asymmetric hydrogenation of conjugated C=C double bonds.^[43]^ Here we give an account of OYE‐catalysed reactions that are useful in organic chemistry.

**Figure 5:**
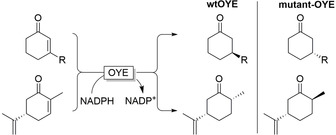
Examples of OYE‐directed evolution to access opposite enantiomers.

OYEs are flavin mononucleotide (FMN)‐dependent oxidoreductases that catalyse the reduction of α,β‐unsaturated compounds with an electron‐withdrawing group (EWG) comprising either an aldehyde, ketone, acid, ester, nitro or nitrile group. The appeal of OYEs in synthetic chemistry is their ability for asymmetric hydrogenation, providing routes to chiral α‐substituted and/or β‐substituted compounds, often with exquisite enantioselectivity. As the hydrogenation occurs in a *trans* fashion, the products are complementary to the well‐established non‐enzymatic *cis* hydrogenation.^[44]^ Moreover, directed evolution studies have been successful in producing enzymes with opposite facial selection of the flavin (“flipped” binding mode), yielding the opposite enantiomers (Figure 5).^[45]^

**Figure 6:**
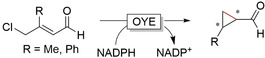
Asymmetric reductive carbocyclisation using engineered OYEs (active site proton donating Tyr replaced with Phe or Trp). Additional electrophilic group either Cl or Br.

Apart from the ever‐increasing substrate scope of OYEs due to new compound screening as well as directed evolution of the catalysts, new reactivities have also been realised. The reductive cyclization by mutant OYEs of α,β‐unsaturated aldehydes and ketones containing an additional electrophilic group via an enolate intermediate enables the enantioselective synthesis of chiral cyclopropanes (Figure 6).^[46]^

**Figure 7:**
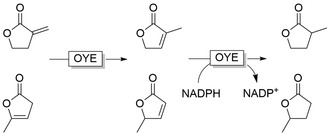
Sequential NAD(P)H‐independent isomerisation and NAD(P)H‐dependent reduction of unsaturated lactones by OYEs.

OYEs have also been reported to perform nicotinamide‐independent C=C bond isomerisation, of both endo‐ and exocyclic double bonds. This allows the subsequent reduction of the conjugated double bonds,^[47]^ as proven for α,β‐unsaturated γ‐butyrolactones and the conversion of α‐angelica lactone to γ‐valerolactone (Figure 7 bottom).^[48]^

Due to the sometimes strict stereoselectivity of OYEs, divergent reduction of stereoisomers, or unreactive stereoisomers, are often encountered when starting with isomeric mixtures. Hartwig, Zhao and co‐workers recently demonstrated stereoconvergent reduction of *E*/*Z* mixtures of alkenes, such as aryl diesters, through a cooperative chemoenzymatic reaction that combines photocatalysts for isomerisation with OYEs for subsequent reduction, allowing stereoconvergent reduction of *E*/*Z* mixtures of alkenes.^[49]^

**Figure 8:**
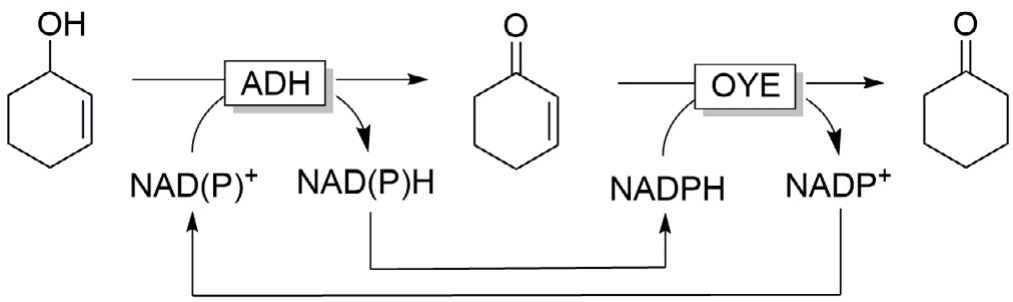
Redox‐neutral conversion of cyclohexenol to cyclohexanone.

OYEs have often been paired with ADHs to allow for redox‐neutral cascade reactions (hydrogen‐borrowing cascades) without the requirement of an external electron donor. Early examples include the redox isomerisation between cyclohexenol and cyclohexanone (Figure 8).^[50]^

**Figure 9:**
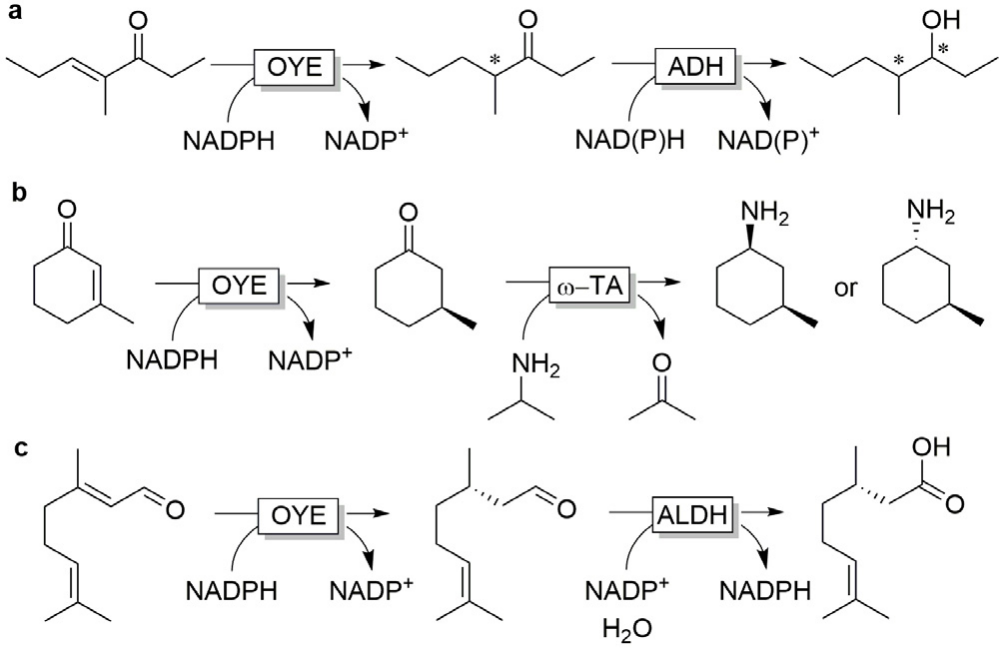
Selected examples of conjugated C=C double‐bond reduction by OYEs coupled to a) alcohol dehydrogenase (ADH) reduction to form chiral alcohols, b) transaminases (TA) for chiral amines, and c) aldehyde dehydrogenases (ALDH) for chiral carboxylic acids.

Despite their simplicity, the combination of OYEs and ADHs, when both used in the reductive direction, allows for the formation of multiple stereoisomers depending on their enantioselectivity, as was recently demonstrated for the synthesis of all four stereoisomers of 4‐methylheptan‐3‐ol (Figure 9 a)^[51]^ and the multiple stereoisomers of dihydrocarveol.^[52]^

OYEs have also been paired with transaminases in sequential reactions to obtain diastereomerically enriched amine derivatives (Figure 9 b)^[53]^ as well as with aldehyde dehydrogenase (ALDH) for the synthesis of chiral α‐ or β‐substituted carboxylic acids from substituted α,β‐unsaturated aldehydes (Figure 9 c).^[54]^ ALDH pairing, similar to ADH coupling, allows for redox‐neutral cascades.

Also cascades combining OYEs with Baeyer–Villiger monooxygenases (BVMOs), giving access to a broad range of lactone products, are being explored intensively now.^[55]^

**Figure 10:**
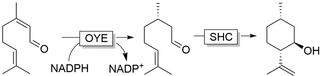
OYE‐mediated reduction of citronellal to citral with concomitant cyclization to (−)‐*iso*‐isopulegol via squalene hopene cyclase (SHC).

Peters and Buller recently reported a linear cascade^[56]^ employing the OYE YqjM from *Bacillus subtilis* for the conversion of citral to citronellal, which in turn was cyclised to (−)‐*iso*‐isopulegol via a mutant of squalene hopene cyclase (SHC) from *Alicyclobacillus acidocaldarius* (Figure 10). This enantiopure product can serve as a precursor for isomenthol or (iso)menthones via chemoenzymatic routes,^[57]^ or possibly further functionalised by the above‐mentioned cascades employing ADHs and BVMOs.

In addition to new OYE homologues becoming available through simple sequence similarity searches, new classes of enzymes with ene reductase activities can be discovered from structural databases. For example, *Ph*ENR and *Tt*ENR (annotated as a FMN‐binding protein and as a putative styrene monooxygenase, respectively) were revealed to possess promiscuous ene reduction activity.^[58]^ Importantly, the mirror symmetry of the active sites of these two enzymes, as compared to OYEs, allowed for the production of the opposite enantiomers due to inverted stereopreference.

**Figure 11:**
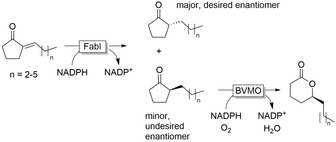
FabI‐catalysed reduction of alkylidenecyclopentanones combined with a BVMO‐catalysed “polishing” reaction, wherein the BVMO removes the undesired enantiomer via oxidative kinetic resolution.

Ene reductase activity has also been observed with Zn‐independent medium‐chain dehydrogenases/reductases (MDRs) such as alkenal/alkenone oxidoreductase^[59]^ and other members of this subfamily.^[60]^ Other classes of enzymes such as the short‐chain dehydrogenase/reductase (SDRs), typically known for their activity as ADHs, have been identified to possess ene ‐reductase activity.^[61]^ Unlike the OYEs, SDRs and MDRs are flavin independent, with the direct transfer of hydride from NAD(P)H. The enoyl acyl carrier protein reductase (FabI), known for its activity to reduce the C=C double bond in an enoyl moiety covalently linked to an acyl carrier protein (ACP), has also been shown to accept simple 2‐alkylidenecyclopentanones.^[62]^ The C=C bond reduction, however, was not highly enantioselective; this could be solved by oxidising the undesired enantiomer in a BVMO‐catalysed kinetic resolution (Figure 11).

Most OYEs utilise NADPH as the preferred reductant. Improving the reactivity with the cheaper NADH via protein engineering is possible.^[63]^ Despite readily available dehydrogenases for NAD(P)^+^ reduction (such as GDH, ADH, phosphite DH, etc.), the past decade has seen the drive for alternative NAD(P)H regeneration systems, such as the electrochemical reduction of NADPH via different mediators,^[26d]^ alternative hydride sources,^[64]^ as well as cofactor‐free reduction of ERs. Non‐enzymatic reduction of FMN, with reducing equivalents originating from formate, through [Cp*Rh(bpy)(H_2_O)]^2+^,^[65]^ as well as light‐driven (photocatalytic) reduction of FMN^[66]^ or deazaflavins^[67]^ using a sacrificial electron donor such as EDTA, and electrocatalytic reduction of FMN^[68]^ have been demonstrated for OYEs. Likewise, photobiocatalytic reduction of C=C double bonds was shown using OYEs with different light sensitizers such as Rose Bengal^[69]^ and transition metal complexes.^[70]^

**Figure 12:**
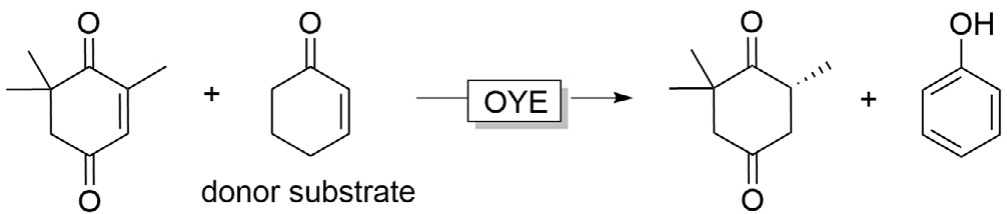
Cofactor (NADPH) independent reduction of ketoisophorone using cyclohexenone as hydride source.

The ability of OYEs to catalyse disproportionation reactions has been exploited to develop NAD(P)H‐independent methods, whereby a cheap co‐substrate serves as the hydride source (Figure 12).^[71]^

**Figure 13:**
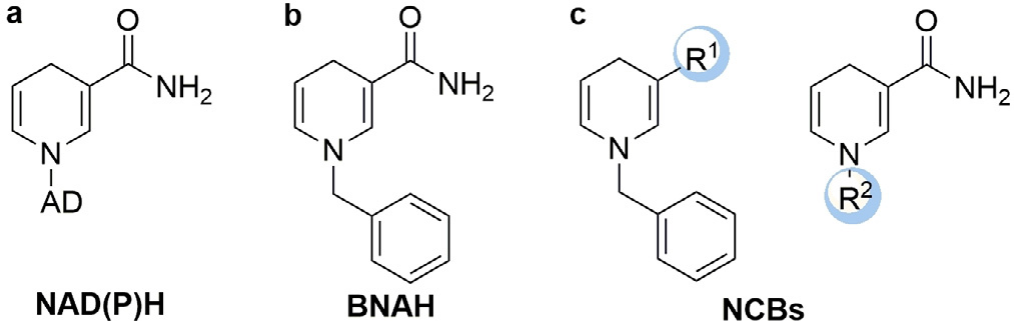
a) Natural cofactor, nicotinamide adenine with dinucleotide (phosphate) (NADH/NADPH), b) typical cofactor analogue 1‐benzyl‐1,4‐dihydronicotinamide (BNAH), and c) substituted nicotinamide cofactor biomimetics (NCBs).

More recently, NAD(P)H cofactor biomimetics (NCBs) have been demonstrated to efficiently (even better than their natural physiological counterparts) drive the reduction of OYEs (Figure 13).^[72]^

Until now, relatively few examples of preparative‐scale ER‐mediated reductions have been reported (Table 2).

**Table 2:** Selection of preparative‐scale ER‐catalysed stereoselective reductions. 
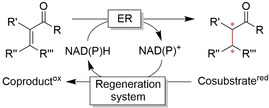

Product	Catalyst	Substrate conc.	Ref.
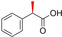	GOx‐ER (0.6 g *E. coli* cell‐free crude extract)	10 g L^−1^ (67 mm), >99 % conv. (85 % yield), >99 % *ee* (*R*)	[73]
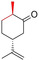	NostocER1 mutant (30.5–33.7 *E. coli* gCDW L^−1^)	45 g L^−1^ (300 mm), 95.6 % yield, 95.4 % *de*	[74]
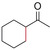	NCR (0.9 g *E. coli* cell‐free crude extract)	100 g L^−1^ (805 mm), >99 % conv.	[73]
	ENE‐101 (1 g *E. coli* cell‐free crude extract)	257 g L^−1^, 9.34 g (1.5 m), >90 % conv.	[75]
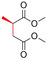	ERED‐04/ER‐104 (70 g cell paste)	70 g, 98 % conv.	[76]
	ENE‐102 (1 g *E. coli* cell‐free crude extract)	161 g L^−1^, 5.9 g (0.73 m), >99 % conv.	[75]
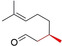	OYE2p (40 U purified)	30 g L^−1^ (0.2 m), >98 % conv. (87 % yield), 89 % *ee* (*R*)	[77]

GOx‐ER: ER from *Gluconobacter oxydans*; NCR: ER from *Zymomonas mobilis*; ERED‐4/ER‐104: from selectAZyme screening kit; ENE‐101/2: from Johnson Matthey's ER collection; OYE2p: ER from *Saccharomyces cerevisiae* YJM1341; NostocER1: NADH‐accepting ER mutant from *Nostoc* sp. PCC7120.

Finally, Bashiri, Colin, Scott, and Greenings made major recent contributions to the F_420_ cofactor biosynthetic pathways, which enabled its application in biocatalysis.^[78]^ The recent discovery and characterisation of ene reductase activity by Fraaije on F_420_H_2_‐dependent reductases (FDRs) has opened the door to new types of catalysts for asymmetric hydrogenations with opposite stereoselectivity to those of OYEs.^[79]^ Unlike OYEs, FDRs use deazaflavin cofactors and can perform *cis*‐hydrogenation.^[80]^

### 2.2 C≡C Triple Bonds

**Figure 14:**
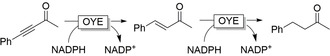
OYE reduction of 4‐phenyl‐3‐butyn‐2‐one via the *trans*‐alkene to 4‐phenyl‐2‐butanone.

To date, the only biocatalytic alkyne reduction was that of 4‐phenyl‐3‐butyn‐2‐one by ERs from the OYE family to form only the corresponding *trans*‐alkene isomer.^[81]^ As these OYEs also reduce C=C double bonds, the alkene intermediate was simultaneously reduced to 4‐phenyl‐2‐butanone (Figure 14).

### 2.3 Aromatic Systems

The Birch reduction is particularly useful in synthetic organic chemistry, as this reaction gives access to cyclic dienes from an aromatic benzene ring. A typical Birch reduction reaction of aromatic rings is carried out under harsh and dangerous conditions: liquid ammonia with sodium, lithium or potassium and an alcohol, such as ethanol and *tert*‐butanol. Alternatively, some tungsten‐containing enzymes have been reported to be capable of catalysing Birch‐like reduction reactions.^[82]^

**Figure 15:**
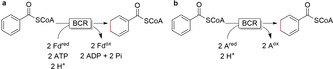
Benzoyl‐coenzyme A reductases (BCRs)‐catalysed reduction of an activated benzene ring; a) Catalysed by ATP‐ and Fd‐dependent class I BCR, b) Catalysed by ATP‐independent class II BCR, accepting a range of alternative electron donor (A^red^). ATP: adenosine triphosphate; ADP: adenosine diphosphate; A^ox^: variable artificial one‐electron acceptor; Fd: ferredoxin.

Enzymatic Birch reduction reactions are reversible.^[83]^ Benzoyl‐coenzyme A reductases (BCRs) catalyse the reduction of an activated benzene via ferredoxin and use either adenosine triphosphate (ATP, class I BCR, Figure 15 a) or a variable electron acceptor (class II BCR, Figure 15 b). The reaction proceeds with alternate single electron/proton transfer steps to the aromatic ring to generate the cyclohexadiene product.^[83]^ These enzymes should definitely be further explored for their synthetic potential in the future!

**Figure 16:**
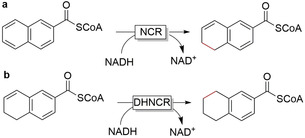
Enzymatic reduction of naphthalene and naphthoyl derivatives. a) NCR‐catalysed regioselective reduction, b) DHNCR‐catalysed reduction. NCR: 2‐naphthoyl‐coenzyme A reductase; DHNCR: 5,6‐dihydro‐2‐naphthoyl‐coenzyme A reductase.

The enzymatic reduction of naphthalene derivatives was only recently characterised by Boll and co‐workers with 2‐naphthoyl‐coenzyme A reductases (NCR), including a 5,6‐dihydro‐2‐naphthoyl coenzyme A reductase (DHNCR) (Figure 16).^[84]^ These enzymes were found to be flavin‐dependent members of the OYE‐like family. Essentially, they catalyse the enantioselective reduction of activated alkenes with NAD(P)H as a hydride source but through a different type of mechanism than OYE to overcome the redox potential barrier of the naphthoyl‐ring system in the substrate.^[84c]^ Other reductase enzymes from the short‐chain reductase family also reduce aromatic compounds such as phloroglucinol, polyhydroxylated naphthalenes and anthracenes (anthrahydroquinones).^[85]^ The potential of these enzymes, like the BCRs, remains to be tapped for applied biocatalysis!

## 3 Reduction of C−X bonds

**Figure 17:**
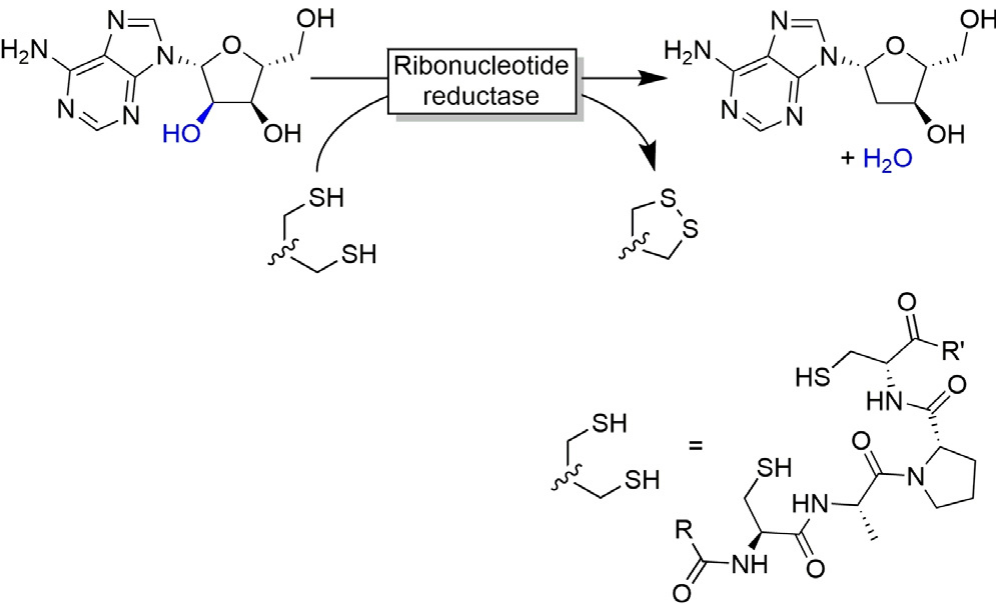
Ribonucleotide reductase‐catalysed reductive deoxygenation reaction.

Nature offers enzymes capable of reductive deoxygenation of C−OH groups.^[86]^ In reactions catalysed by ribonucleotide reductase, cobalamin‐generated thiyl radicals perform H‐atom abstraction and overall reduction of the diol (Figure 17); thioredoxin serves as reductant.^[87]^

This appealing reaction, however, has so far not received much interest in the context of preparative biocatalysis. This is also true for further cobalamin (vitamin B_12_)‐dependent enzymes such as a range of dehalogenases catalysing the selective reductive dehalogenation of a broad range of compounds. While the latter are rather well‐known in the context of environmental biotechnology, they are practically unknown in the biocatalysis community.^[88]^

Reductive dehalogenation has been reported with a highly reduced, thermophilic CYP119 from *Sulfolobus solfataricus* ^[89]^ and by using reductive dehalogenases (cobalamin‐dependent) from some dehalogenating microbes such as *Dehalobacter* or *Dehalococcus*.^[90]^ Hence, enzymatic reductive deoxygenations and dehalogenations remain a highly promising, yet to be explored new frontier of biocatalysis!

**Figure 18:**
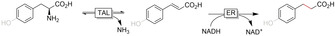
Formal reductive deamination of phenylalanine or tyrosine using a combination of tyrosine amine lyase (TAL) and an ene reductase (ER).

More straightforward are indirect, cascade reactions, which overall entail a reductive deamination or deoxygenation. A cascade comprising amino acid ammonium lyases and ene reductases for example, results in an overall reductive deamination of amino acids (Figure 18).^[91]^

Principally, similar cascades combining C=C hydratases and ene reductases, for example, to convert malic acid (or tartaric acid) into succinic acid as found in the citric acid cycle are possible. Of course the scope of these deamination and deoxygenation reactions is limited due to the selectivity of the biocatalysts available. However, the recent success of protein engineering gives rise to the hope that this may change if properly addressed.

**Figure 19:**
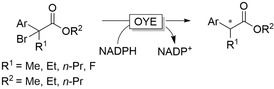
Enantioselective (radical) dehalogenation of α‐bromoesters using OYEs.

OYEs have also been shown to catalyse the enantioselective radical dehalogenation of α‐bromoesters through a single‐electron reduction from the flavin hydroquinone and subsequent hydrogen transfer from the semiquinone (Figure 19).^[92]^ Reductive deacetoxylation with light activation of ene reductases was also demonstrated.^[93]^

## 4 Reduction of C=O Bonds

### 4.1 Reduction of Aldehydes

**Figure 20:**
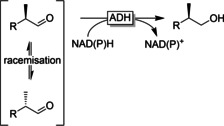
Reductive dynamic kinetic resolution of racemic, α‐substituted aldehydes.

Chemoselective reduction of the aldehyde group in the presence of further reducible functionalities such as ketones and C=C bonds is possible using selective ADHs.^[94]^ Exploiting the enantioselectivity of ADHs appears awkward for the reduction of aldehydes. However, the well‐defined macromolecular architecture of the enzymes’ active sites also allows for the discrimination of distant stereocenters. With α‐substituted aldehydes, dynamic kinetic resolutions are also possible by exploiting the racemising keto–enol equilibrium (Figure 20).^[95]^ With activated, enolisable aldehydes (e.g. profene aldehydes) the racemisation occurs in situ, while in the case of less activated aldehydes the racemisation has to take place ex situ.

**Figure 21:**
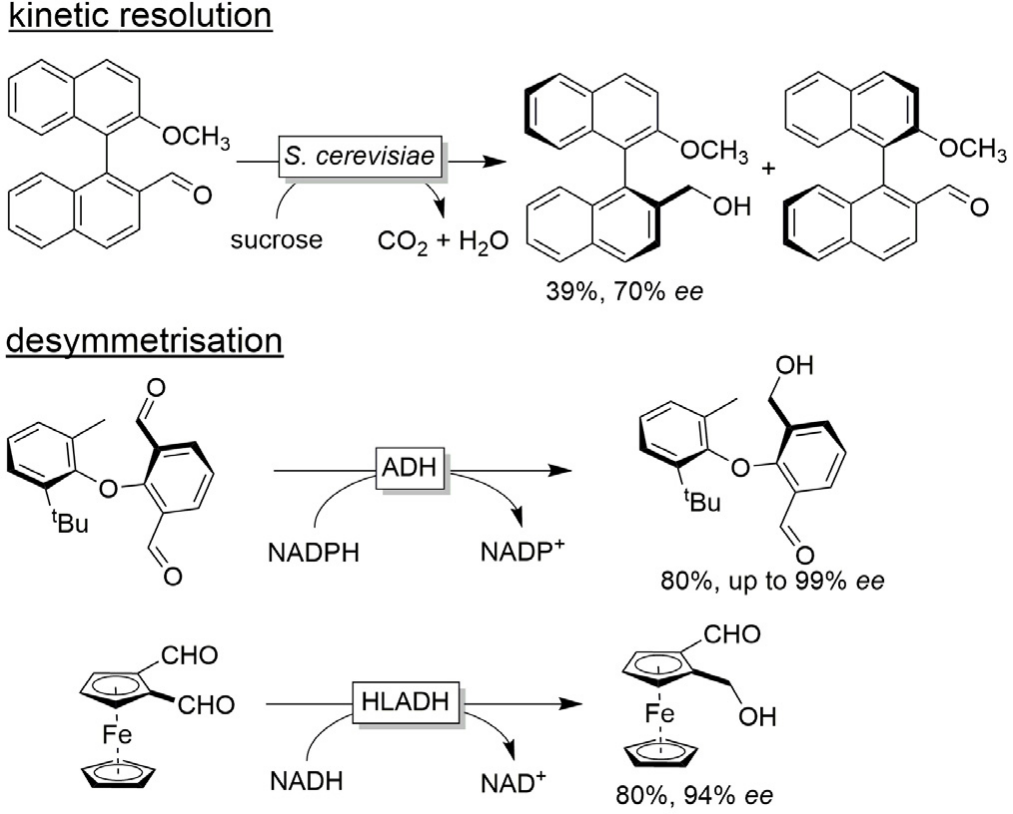
Examples of ADH‐catalysed kinetic resolutions and desymmetrisation reactions of molecules lacking point chirality.

Also, less common (pro)chiral molecules have been investigated such as in the kinetic resolution of atropisomeric binaphthyls^[96]^ or the desymmetrisation of diarylethers^[97]^ and organometallic sandwich complexes (Figure 21).^[98]^

Finally, the recently rediscovered biocatalytic Cannizzaro reaction should be mentioned, in which racemic profene aldehydes could be disproportionated into enantiopure alcohols and carboxylic acids.^[99]^

### 4.2 Reduction of Ketones

#### 4.2.1 …To Alcohols

ADHs are the catalysts of choice for the stereoselective reduction of ketones to alcohols. Systems utilising other enzyme classes such as ene reductases^[100]^ or Baeyer–Villiger monooxygenases^[101]^ are still at an early stage. ADHs, however, have been investigated for more than four decades now and a vast variety of enzymes (and variants) are available. Hence, also the industrial interest in ADH catalysis is steadily growing.[^10^, ^102^, ^103^] A selection of ADH‐derived alcohols shown in Table 3 illustrates how widespread this technology is today, also on an industrial scale. Pleasingly, ADH‐catalysed ketoreduction reactions are increasingly performed at significant substrate loadings, resulting in preparatively relevant product concentrations in the molar range (Table 3). Neat reaction conditions and 2LPSs are employed, thereby overcoming solubility issues for hydrophobic reagents.

**Table 3:** Selection of ADH‐catalysed stereoselective ketone reductions. 
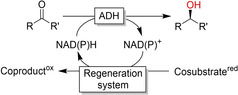

Product	Catalyst	Product conc.	Ref.
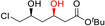	*Ss*SCR as whole *E. coli* cells (40 g L^−1^)	500 g L^−1^	[104]
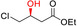	*Aa*ADH	660 g L^−1^	[105]
	*Rp*CR	700 g L^−1^	[106]
	*Sc*CR	255 g L^−1^	[107]
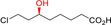	*Rh*CR	440 g L^−1^	[108]
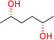	*Tt*HBD in *E. coli*	170 mm	[109]
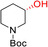	KR‐110 (commercial)	100 g L^−1^	[33]
	*Ch*KRED03	200 g L^−1^	[110]
	*Sc*KRED in *E. coli*	240 g L^−1^	[111]
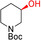	*Km*CR	400 g L^−1^	[112]
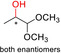	KRED (commercial)	250 g L^−1^	[113]
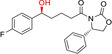	CR in *E. coli* (11 g L^−1^)	150 g L^−1^	[114]
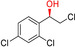	*Schs*CR (mutant) in *E. coli*	268 g L^−1^	[115]

*Ss*SCR: short‐chain ADH from *Sporobolomyces salmonicolor* AKU442 (mutant); *Aa*ADH: ADH from *Acetobacter aceti*; *Rp*CR: carbonyl reductase from *Rhodococcus pyridinivorans*; *Rh*CR: *Rhodococcus* sp. ECU1014; *Tt*HBD: 3‐hydroxybutyryl‐CoA dehydrogenase from *Thermus thermophilus* HB8; *Sc*CR: carbonyl reductase from *Streptomyces coelicolor* (engineered); *Schs*CR: carbonyl reductase from *Scheffersomyces stipitis* CBS 6045; *Ch*KRED03: ketoreductase from *Chryseobacterium* sp. CA49; *Km*CR: carbonyl reductase from *Kluyveromyces marxianus* ATCC 748; *Sc*KRED: ketoreductase from *Saccharomyces cerevisiae*;

Having solved the basic impediments such as cofactor regeneration, low substrate loadings and narrow substrate scope, current academic research focusses on extending ADH reactions. Enolisable racemic starting materials, for example, are a popular subject, as they allow for the generation of two chiral centres in one step.^[116]^ An interesting example of the stereoselective reduction of α‐substituted ketones under enolising conditions was reported by Xu, Kosjek and co‐workers as a key step in the synthesis of Vibegron.^[117]^ A ketoreductase was evolved to meet the temperature and pH requirements of the epimerisation reaction as well as to become more stereoselective. After only three rounds of directed evolution, a KRED mutant was obtained meeting the requirements (more than 95 % conversion at pH 10, *T*=45 °C, [substrate]=50 g L^−1^ and 1 % (w/w) of the engineered KRED mutant), thereby impressively demonstrating the potential of protein engineering to tailor enzyme properties.

**Figure 22:**
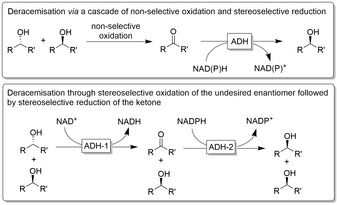
Deracemisation of alcohols using stereoselective ADH‐catalysed reduction of an intermediate ketone as the key step.

Another sustained trend in academic research is the embedding of stereoselective ketoreduction into more complex reaction schemes. Multistep deracemisation of alcohols is just one example for this.^[118]^ Typically, a racemic alcohol starting material is transformed into the prochiral ketone (either by non‐selective, full oxidation or by kinetic oxidative resolution) followed by stereoselective ADH‐catalysed reduction of the ketone (Figure 22).

Deracemisation of alcohols using non‐selective, non‐enzymatic oxidants typically make use of organometallic^[119]^ or organocatalytic^[120]^ oxidants. Despite its simplicity, this approach is hampered by the need for a temporal separation of both steps to avoid futile oxidation of the product alcohol formed during the stereoselective reduction step. Using two stereo‐ and cofactor‐complementary ADHs circumvents this limitation, as the oxidative kinetic resolution and the stereoselective reduction of the intermediate ketone can be performed in a one‐pot, one‐step cascade. Since the pioneering work by Kroutil and co‐workers,^[121]^ this approach has found widespread applications.^[118]^

**Figure 23:**
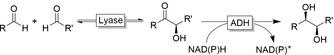
Bi‐enzymatic cascade comprising lyase‐catalysed C−C bond formation (acyloin) followed by stereoselective carbonyl reduction yielding the diol.

An interesting example for the embedding of stereoselective ketoreduction is the combination with a precedent carboligation step catalysed by aldehyde lyases (such as benzaldehyde lyase of pyruvate decarboxylase), enabling the synthesis of (a)symmetric diols from two aldehydes (Figure 23).[^29a^, ^122^]

#### 4.2.2 …To Amines

Amino acid dehydrogenases (AaDHs) were the first enzymes used for the reductive amination of carbonyl groups (of α‐keto acids). Today, a range of industrial‐scale syntheses of unnatural α‐amino acids using AaDHs have been established.[^29h^, ^123^] A broader product scope of AaDH‐applications is hampered by their limitation to α‐keto acids as starting material and their high selectivity towards l‐amino acids, which in principle can be addressed by protein engineering.^[124]^

Bommarius and co‐workers engineered an AaDH into an amine dehydrogenase (AmDH) to catalyse the reductive amination of ketones as well.^[125]^ Further protein engineering by the groups of Mutti^[126]^ and Xu^[127]^ led to further enlargement of the AmDH substrate scope to aromatic substrates and primary amines.

Recently, also native AmDHs have been discovered.^[128]^ Still in the infancy of development, native and engineered AmDHs are definitely promising enzymes to catalyse asymmetric reductive amination.^[129]^

Following the AaDHs, transaminases (TAs) are gaining ground in the synthesis of chiral amines. With more than 20 years of intensive research, issues such as limited substrate scope, substrate/product inhibition or poor stability of the biocatalysts can be solved efficiently via protein engineering or reaction engineering.[^19e^, ^130^] Transaminase‐catalysed reactions are constantly gaining popularity especially in the pharmaceutical industry.^[131]^ Table 4 gives a representative, but by no means exhaustive, overview over several TA‐catalysed reductive aminations.

**Table 4:** Selection of chiral amines obtained via TA‐catalysed reductive amination of ketones. 
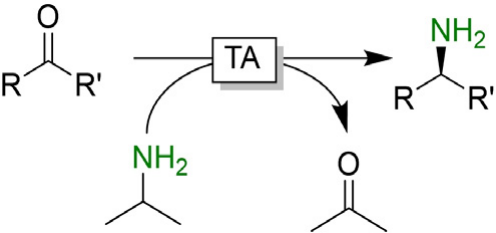

Product	Catalyst	Remark	R.ef
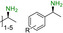	*BmTA, AdTA, AsTA, CvTA*		[132]
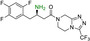	ATA‐117; highly engineered	200 g L^−1^	[133]
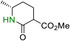	ATA‐117	5 kg scale	[134]
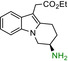	ATA‐117		[134]
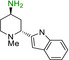	ATA‐036	two stereocenters in one step	[135]
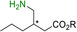	*Vf*TA	both enantiomers, DKR of the racemic starting aldehyde	[136]

*Bm*TA: ω‐TA from *Bacillus megaterium*; *Ad*TA: ω‐TA from *Alcaligenes denitrificans*; *A*sTA: ω‐TA from *Alcaligenes* species; *Cv*TA: ω‐TA from *Chromobacterium violaceum*; ATA‐117: ω‐TA from *Arthrobacter* sp.; ATA‐036: commercially available ω‐TA; *Vf*TA: ω‐TA from *Vibrio fluvialis*.

**Figure 24:**
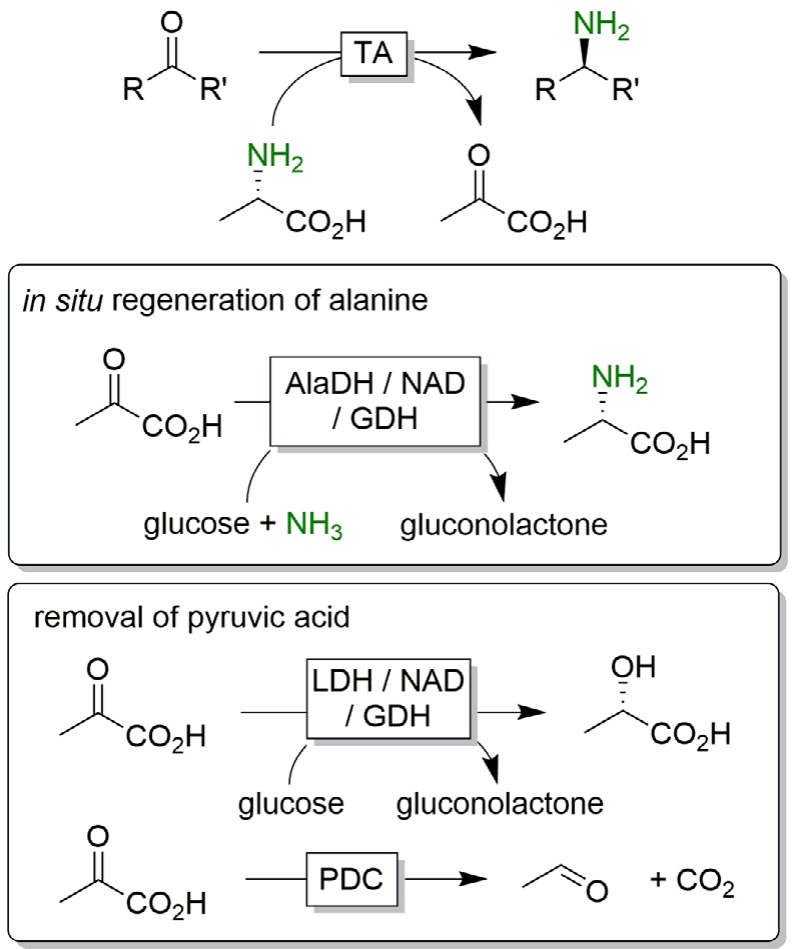
Selection of equilibrium displacement methods for alanine‐driven reductive aminations using transaminases (TAs). AlaDH: alanine dehydrogenase; GDH: glucose dehydrogenase; LDH: lactate dehydrogenase; PDC: pyruvate decarboxylase.

**Figure 25:**
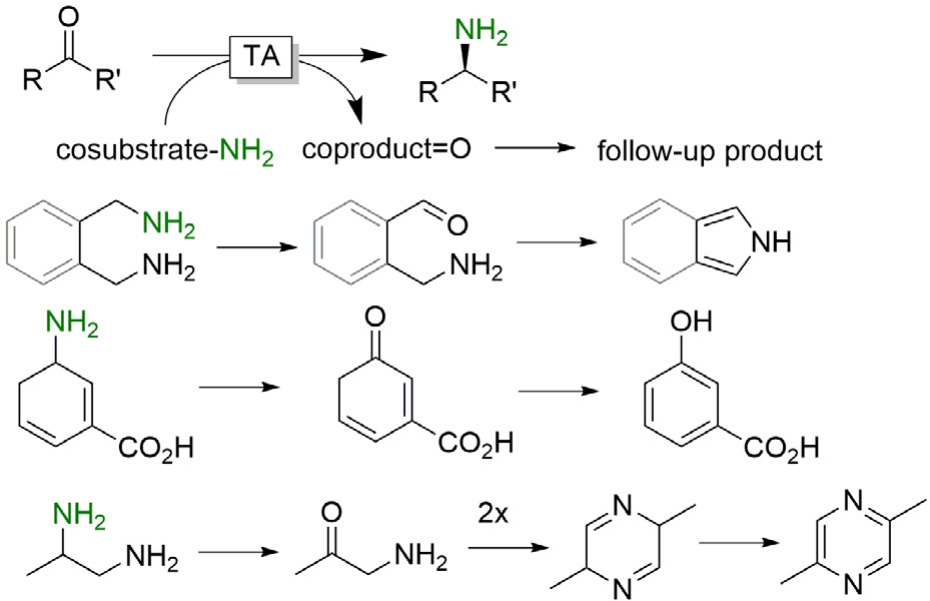
Examples of “smart cosubstrates” for equilibrium displacement in TA‐catalysed reductive aminations through rearrangement to stable aromatic by‐products.

One of the issues that have to be met using TA‐catalysed reductive aminations is the usually unfavourable thermodynamic equilibrium of the reaction. Using the amine donor (such as isopropylamine, alanine or 1‐phenylethylamine) in excess is feasible but leads to further issues in downstream processing and is unattractive from an environmental point of view. The in situ removal of the (undesired) side product (e.g. acetone) is also an option.^[137]^ With this in mind, extensive research has been devoted to shift the equilibrium in the desired direction. The two main approaches are (enzymatic) degradation of the side product (especially of pyruvic acid, Figure 24) and using “smart amine donors” (Figure 25).

To avoid the accumulation of pyruvate as a stoichiometric coproduct, several enzymatic systems have been designed. Simply reducing pyruvate to lactate by lactate dehydrogenase in combination with in situ NADH regeneration is widely used.^[138]^ Similarly, pyruvate decarboxylase efficiently decomposes pyruvate into two volatile products (acetaldehyde and CO_2_) displacing the equilibrium.^[139]^ More elegantly, pyruvate can also be recycled back into alanine, thereby using the costly alanine in catalytic amounts only.[^132a^, ^138b^]

In addition to these enzymatic methods, a range of “smart cosubstrate” approaches have been developed, wherein the TA coproduct spontaneously rearranges into another stable (mostly aromatic) product (Figure 25).[^28b^, ^140^]

**Figure 26:**
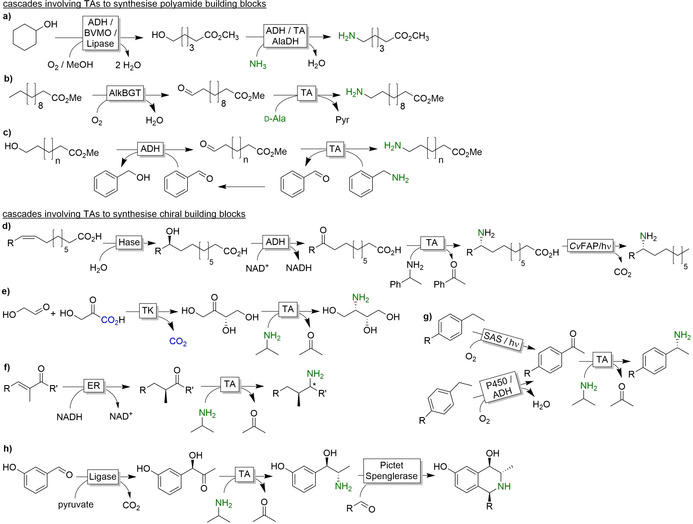
Examples of cascades involving TA‐catalysed stereoselective reductive amination. a) Starting from cyclohexanol yielding ω‐hydroxy hexanoic acid, which is transformed into ω‐amino hexanoic acid;^[147a]^ b,c) starting from fatty acids involving ω‐hydroxylation;[^145a^, ^147b^, ^147c^] d) starting from unsaturated fatty acids involving hydration of the C=C double bond followed by oxidation and reductive amination and decarboxylation;^[145]^ e) C−C bond formation prior to reductive amination;^[143]^ f) combined with a stereoselective C=C bond reduction^[53]^ g) in situ oxyfunctionalisation;^[146]^ h) following in situ C−C bond formation.^[144a]^ ADH: alcohol dehydrogenase; BVMO: Baeyer–Villiger monooxygenase; TA: transaminase; AlaDH: alanine dehydrogenase; AlkBGT: alkane monooxygenase; Hase: hydratase; *Cv*FAP: photoactivated fatty acid decarboxylase; ER: ene reductase; TK: transketolase; SAS: sodium anthraquinone sulfonate.

From an early stage on, TAs have been used not only in stand‐alone reductive aminations but also as part of more complex cascades. Classical examples comprise cascades for the synthesis of optically pure amines from racemic alcohols^[141]^ and amines.^[142]^ In addition, some very interesting cascades building complex chiral amines from simple starting materials have been developed. These comprise preceding C−C bond formations catalysed by transketolases^[143]^ or lyases (Figure 26 e,h).^[144]^ α‐Substituted amines are accessible from enones by combining ene reductases and TAs (Figure 26 f).^[53]^ Attention has been paid to the in situ generation of the carbonyl group either from C=C double bonds via a cascade of hydratase‐catalysed water addition and ADH‐catalysed oxidation to the ketone (Figure 26 d),^[145]^ or from sp^3^‐hybridzed C−H groups via enzymatic or photochemical oxyfunctionalisation (Figure 26 g).^[146]^

More recently, TAs have been evaluated as catalysts in the synthesis of ω‐amino acid(esters) as polyamide building blocks (Figure 26 a–c).[^145a^, ^147^] Considering the bulk character of such products, this underlines the ambitions of TA technology to leave the chiral products‐only niche!

In general, transaminase‐catalysed reductive amination has become an integral component of biocatalysis and has become a viable alternative to classical non‐enzymatic methods!

## 5 Reduction of C=N Bonds

**Figure 27:**
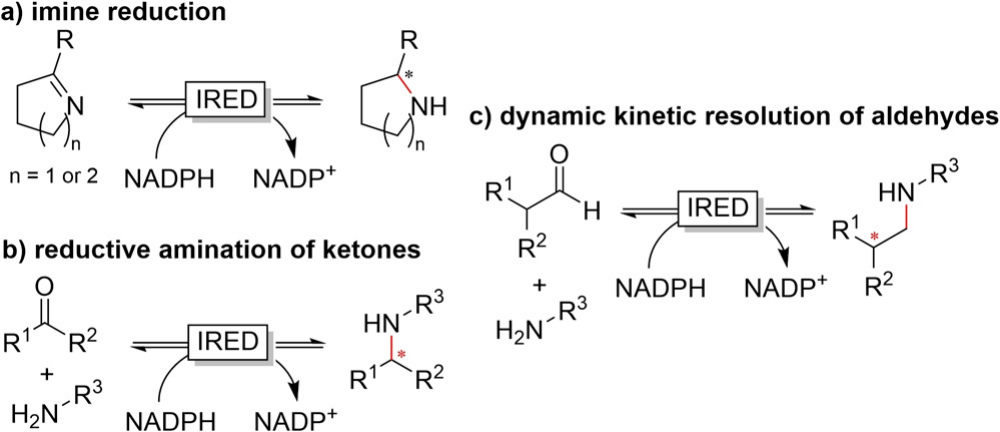
IRED‐catalysed reactions starting from an imine (a), reductive amination from ketone and amine with in situ imine formation (b), dynamic kinetic resolution of aldehydes (c).

Since 2010 imine reductases (IREDs) have been used in biocatalysis to catalyse the reduction of imines.^[148]^ Their development has been exponentially increasing thanks to bioinformatics, mining available protein databases, with several practical reviews already describing various processes as testimony.^[149]^ IREDs selectively catalyse the asymmetric reduction of (prochiral) imines and iminium ions to the corresponding secondary and tertiary (chiral) amines. There are various reaction scheme scenarios in which IREDs can be used: starting directly from an imine (including cyclic and exocyclic) as a substrate, or with in situ formation of the imine in solution from an amine and ketone,^[21a]^ essentially catalysing reductive amination (Figure 27). With few exceptions,^[150]^ the current IREDs are NADPH‐dependent.

**Figure 28:**
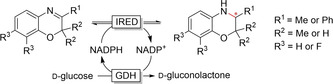
IRED‐catalysed synthesis of benzoxazines.

New IREDS have been continuously identified and characterised the past few years,^[151]^ and IREDs have been shown to accept quite a range of imines formed from ketones and amines.^[152]^ Imine reductase activity has also been found in several short‐chain reductases (SDRs).[^85^, ^153^] Several SDRs are able to reduce C=N, C=O, and C=C double bonds.^[154]^ The group of Gröger demonstrated the use of IRED for the synthesis of benzoxazines (Figure 28).^[155]^

**Figure 29:**
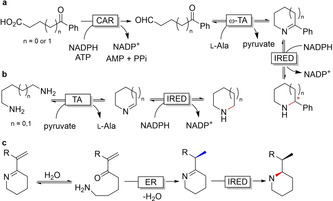
a) Example of an enzymatic cascade with IREDs to obtain chiral piperidines. CAR=carboxylic acid reductase. b,c) Bi‐enzymatic cascade with b) a putrescine transaminase and IRED to obtain piperidines, c) ER and IRED for the selective reduction of enimines to chiral piperidines derivatives.

IREDs have been combined with other enzymes in cascade reactions to produce valuable chiral amine compounds. One example is a three‐enzyme cascade for the production of chiral mono‐ and disubstituted piperidines and pyrrolidines starting from keto acids, with high enantiomeric excess (Figure 29 a).^[156]^ A bi‐enzymatic cascade with putrescine transaminase led to another path towards generating piperidines and pyrrolidines (Figure 29 b).^[157]^ Thorpe et al. further elegantly combined an ER with an IRED to reduce enimines, which are prone to hydrolysis and thus becoming a substrate for ER before the imine is selectively reduced by the IRED (Figure 29 c).^[158]^

**Figure 30:**
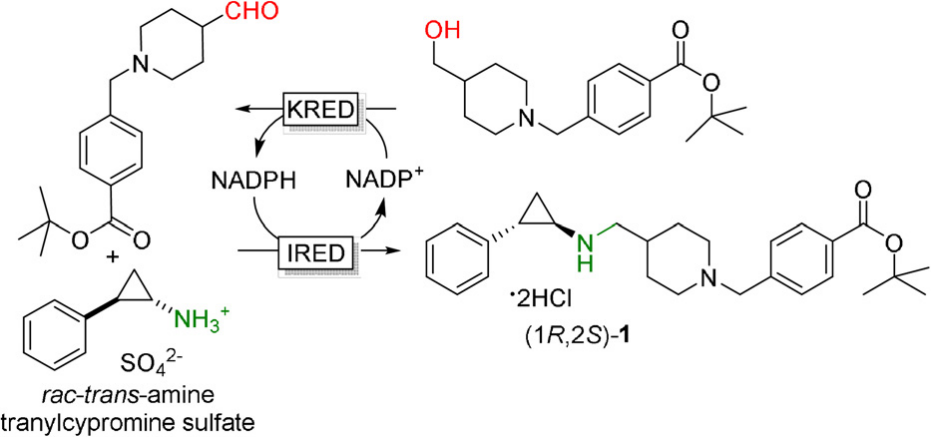
Hydrogen‐borrowing cascade with an IRED‐catalysed reductive amination to provide (1*R*,2*S*)‐**1**, precursor to GSK2879552.

Recently, GSK has demonstrated the use of a highly engineered IRED for the production of one of their target drugs through a hydrogen‐borrowing cascade in which the IRED is coupled to a KRED (Figure 30).^[159]^ Excellent isolated yields of 451 g (84.4 %) and optical purity of the product (99.7 % *ee*) were reported. This example demonstrates that some of the current bottlenecks of IREDs, such as limited substrate scope and the need for a large excess of amine for reductive amination, can be overcome with protein engineering.

Reductive amination can also be catalysed by reductive aminases (RedAm), first introduced with *Asp*RedAm from *Aspergillus oryzae* ^[160]^ by Turner and co‐workers (vide infra*)*.^[161]^ Mutants of *Asp*RedAm were also used for the deracemisation of amines.^[162]^

## 6 Reduction of Carboxylate Groups

The reduction of carboxylic acids is fairly challenging from a thermodynamic point of view and necessitates activation of the carboxylate group. Carboxylic acid reductases (CARs) achieve this activation through the (ATP‐dependent) formation of an enzyme‐bound thioester (Figure S8). The thioester undergoes NADPH‐dependent reduction to the hemi‐thioacetal, which spontaneously eliminates the aldehyde product.^[163]^

CARs have been receiving considerable interest the past few years,[^22^, ^164^] and a broad range of aliphatic and (hetero)aromatic acids have been reported as substrates.[^164b^, ^164c^, ^164g^, ^165^] CARs will possibly play a role in the conversion of natural (renewable) carboxylic acids into chemical building blocks such as diols[^165b^, ^166^] or diamines.^[167]^

**Figure 31:**
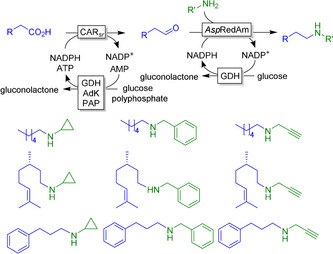
N‐alkylation of carboxylic acids using CAR from *Segniliparus rugosus* (CAR*sr*) and the reductive aminase from *Aspergillus oryzae* (*Asp*RedAM). NADPH regeneration was achieved by the glucose dehydrogenase (GDH) system and ATP regeneration by combining AMP phosphotransferase (PAP) and adenylate kinase (AdK) from *Acinetobacter johnsonii* 210A.

A very interesting cascade was recently reported by Turner and co‐workers using CAR‐activated acids combined with imine reductase‐catalysed formation of the resulting aldehyde to secondary amines (Figure 31).^[161c]^

The CAR mechanism (Figure S8) comprises formation of a mixed anhydride to activate the carboxylate for nucleophilic attack by NADPH. Flitsch and co‐workers demonstrated that other nucleophiles such as amines are feasible giving access to amides.^[168]^

Inspired by the CAR mechanism, we also investigated whether thioesters represent possible substrates for ADHs.^[169]^ Indeed, a range of (chemically synthesized) thioesters were readily converted by some ADHs. We envision that with in situ thioesterification (under non‐aqueous conditions) this may lead to an ATP‐independent reduction system for carboxylic acids.

Besides CARs, aldehyde oxidoreductases are capable of reducing carboxylic acids to aldehydes. These ATP‐independent, molybdenum or tungsten enzymes were reported in the 1980s by Simon and co‐workers for acid reduction.[^42a^, ^42b^, ^42c^, ^170^] More recently, we have demonstrated that the aldehyde oxidoreductases from *Pyrococcus furiosus* can be used for the hydrogenation of carboxylic acids. Interestingly, CO could also be used as reductant.^[2]^

In the context of carboxylate reduction, the growing field of bioelectrocatalytic CO_2_ fixation aiming at closing the anthropogenic carbon cycle should also be mentioned.^[171]^ In essence, if renewable electrical energy is used, overall CO_2_‐negative production systems are possible.

Next to electrochemical reducing power, photochemically provided reducing equivalents are attractive for CO_2_ fixation.^[172]^

## 7 Miscellaneous

Nature's repertoire of reduction catalysts goes far beyond the examples discussed so far. New reduction reactions are constantly being discovered based on screening natural diversity for new reactions or by probing the catalytic promiscuity of known enzymes. (Visible) light irradiation of established enzymes, for example, opens up new synthetic possibilities.[^40a^, ^92^, ^93^, ^173^]

**Figure 32:**
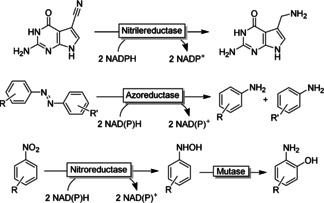
Selection of some miscellaneous biocatalytic reduction reactions.

Figure 32 shows a selection of some “unusual” reductive biotransformations that may become synthetically relevant in the near future.

Nitrile reductases, for example, catalyse the selective reduction of nitriles to the corresponding primary amines, which may be an invaluable addition to existing nitrile chemistry.^[174]^ The catalysts available today, however, are highly specific for their natural substrate, requiring excessive protein engineering to make nitrile reductases practical catalysts for the organic chemist.

Flavin‐dependent azoreductases^[175]^ catalyse the reductive cleavage of diazo compounds into primary aromatic amines. Flavoproteins also mediate the reduction of nitro groups, which is of particular interest for environmental chemistry^[176]^ and can be used for the synthesis of amino phenols from nitroaromatics.^[176c]^

Under reductive conditions, heme‐dependent enzymes catalyse the reduction of azides.^[177]^ The intermediate Fe‐nitrene species, however, can also just hydrolyse prior the next electron‐transfer steps and yield aldehyde or ketone products instead of the fully reduced amine.

**Figure 33:**
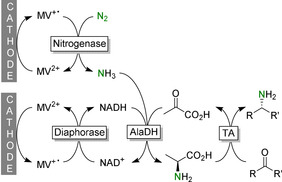
Combined electromicrobial N_2_ fixation and reductive amination.

A very interesting electromicrobial system combining N_2_ fixation with the use of the NH_3_ obtained in a reductive amination was recently reported by Minteer and co‐workers (Figure 33).^[178]^ By using recombinant, whole cells the authors circumvented issues arising from the complex molecular architecture of the nitrogenase. Electrochemical communication of the enzymes with the cathode was established via low‐molecular‐weight mediators such as viologens. Considering the novelty of this system, it performed astonishingly well, accumulating millimolar concentrations of the desired products.

## 8 Summary and Outlook

Biocatalysis is a potent tool for preparative chemistry, especially when it comes to catalytic reduction reactions. The many elegant biocatalytic reduction reactions showcased in this contribution demonstrate that enzymes remain unrivalled when it comes to selectivity. Thus, purer products become available in fewer reaction steps, translating into significant economic and environmental benefits. Today's bioreduction chemistry complements established non‐enzymatic counterparts but we expect that further unravelling of nature's repertoire as well as creative combination of new and engineered enzymatic steps will also enable chemical reduction reactions not yet sought after.

The last decades have seen enormous improvements of some early limitations^[179]^ such as limited substrate scope, poor stability of the catalysts and low substrate loadings.

Today, most industrial examples for bioreduction chemistry are found in the high value‐added fine‐chemical and pharmaceutical sectors but the impressive progress in catalyst efficiency and product titres make production of lower value‐added, bulk products come into reach.

Overall, we see the field of bioreduction catalysis as maturing and rapidly evolving simultaneously. Hence it will remain to be a playground for researchers from different disciplines for years to come.

## Conflict of interest

The authors declare no conflict of interest.

## Biographical Information

*Frank Hollmann studied Chemistry at the University of Bonn (Germany). After his PhD with Andreas Schmid (ETHZ Zurich, Switzerland) and a postdoc with Prof. Manfred T. Reetz (Max‐Planck‐Institut für Kohlenforschung, Germany), he worked for some years at Evonik Industries. In 2008 he joined the biocatalysis group at TU Delft, where he is Professor of Biocatalysis*.

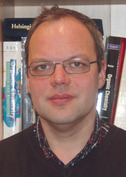

## Biographical Information

*Diederik J. Opperman obtained his PhD in biochemistry at the University of the Free State (South Africa) in 2008. He then conducted postdoctoral research on directed evolution with Prof. Manfred T. Reetz at the Max‐Planck‐Institut für Kohlenforschung (Germany). He is currently an Associate Professor at the University of the Free State (SA) with a research focus on the structure–function relationship of biocatalysts*.

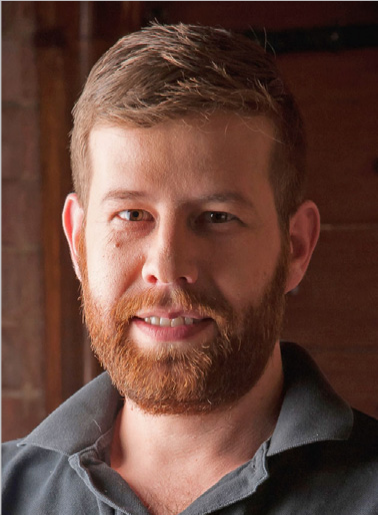

## Biographical Information

*Caroline E. Paul received her Honours BSc and MSc in Biological Chemistry at the University of Toronto and her PhD at the University of Oviedo with Profs. V. Gotor‐Fernández and I. Lavandera. After postdoctoral work as a Marie Curie Fellow at TU Delft, she carried out research on biomimetic cofactors for oxidoreductases with a NWO VENI grant at Wageningen University. Since 2018 she has been Assistant Professor in Biocatalysis at TU Delft*.

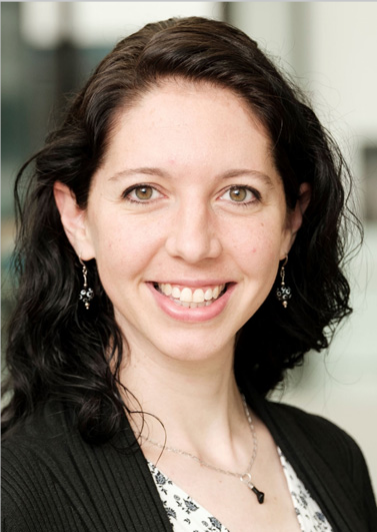

## Supporting information

As a service to our authors and readers, this journal provides supporting information supplied by the authors. Such materials are peer reviewed and may be re‐organized for online delivery, but are not copy‐edited or typeset. Technical support issues arising from supporting information (other than missing files) should be addressed to the authors.

Supplementary
