# Supplementary material for: Biocatalytic Reduction Reactions from a Chemist's Perspective
Source: Angew Chem Int Ed Engl. 2020 Nov 3;60(11):5644–65. doi: 10.1002/anie.202001876 (PMC7983917; doi:10.1002/anie.202001876)
Supplement: Supplementary file 1 — Supplementary [file ANIE-60-5644-s001.pdf]

Angewandte Chemie
Eine Zeitschrift der Gesellschaft Deutscher Chemiker

Supporting Information

Biocatalytic Reduction Reactions from a Chemist's Perspective

Frank Hollmann, Diederik J. Opperman,* and Caroline E. Paul**

anie_202001876_sm_miscellaneous_information.pdf

Contents

1. Chemical structures of the most relevant redox cofactors 2
1.1. NAD(P)H 2
1.2. Flavins 2
2. Catalytic mechanisms of the most relevant reductive enzymes 3
2.1. Alcohol dehydrogenases 3
2.2. Ene reductases 3
2.3. Imine reductases 4
2.4. Transaminases 4
2.5. Amine dehydrogenases 5
2.6. Carboxylic acid reductases 5

1. Chemical structures of the most relevant redox cofactors

1.1. NAD(P)H

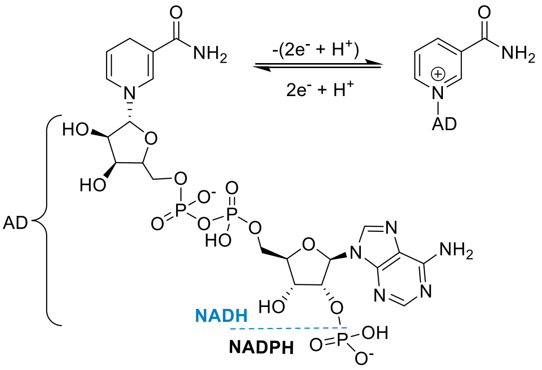

Figure S1. Structure and chemistry of reduced nicotinamide adenine dinucleotide cofactors NADH and NADPH. AD = adenosine diphosphoribose.

1.2. Flavins

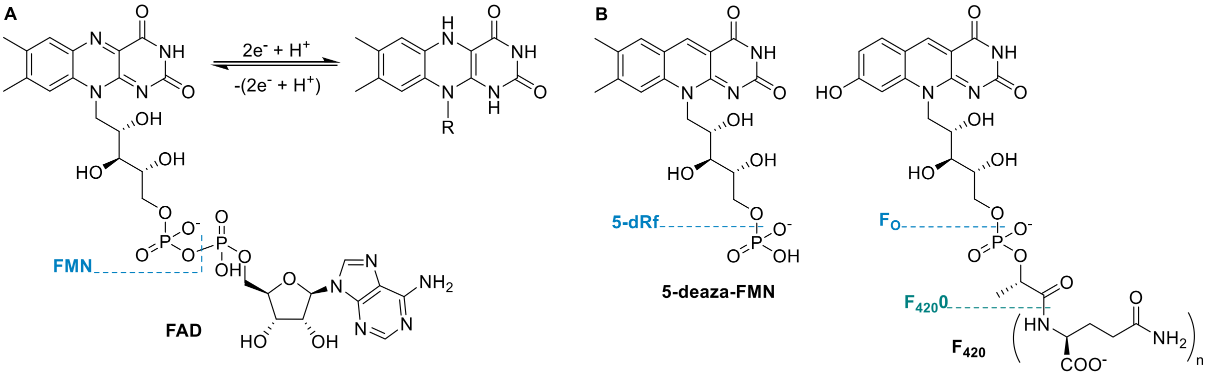

Figure S2. Structure and chemistry of (**A**) flavin mononucleotide (FMN) and flavin adenine dinucleotide (FAD) cofactors, (**B**) 5-deaza-FMN and F_{420} cofactors.

2. Catalytic mechanisms of the most relevant reductive enzymes

2.1. Alcohol dehydrogenases

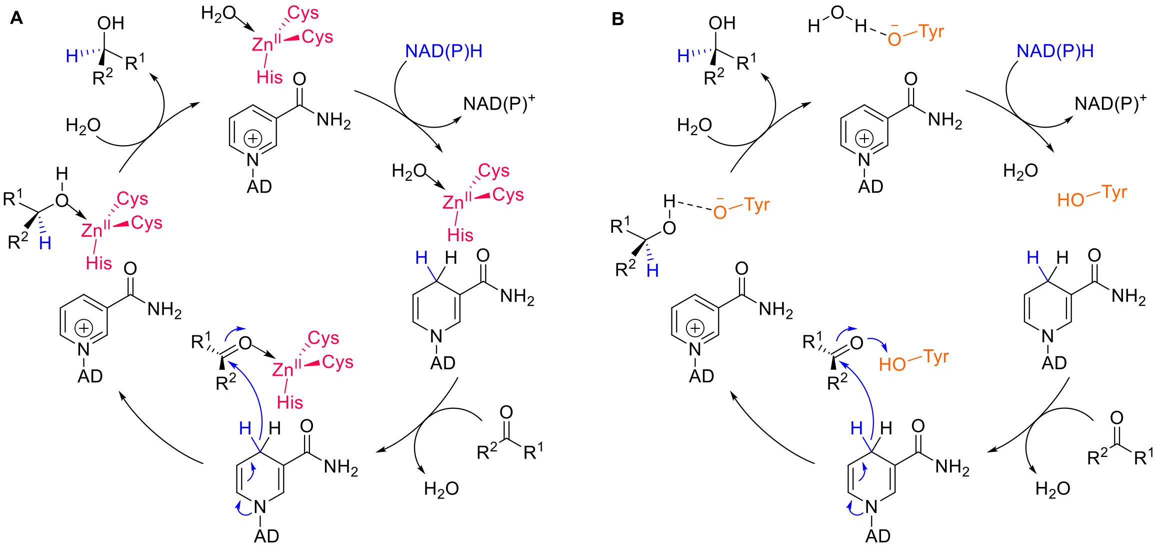

Figure S3. Representative mechanisms of Zn^{2+}-dependent (**A**) MDR-ADH vs (**B**) SDR-ADH-catalysed carbonyl reduction reaction. In the first step, the reduced nicotinamide cofactor binds to the enzyme active site, followed by binding of the carbonyl substrate. After the hydride transfer (yielding the alcohol product and the oxidised nicotinamide cofactor), both products leave the active site (ordered bi-bi mechanism). Each step is reversible. In some enzymes Zn^{2+} is replaced by $Fe^{2+/3+}$. AD = adenosine diphosphoribose.

2.2. Ene reductases

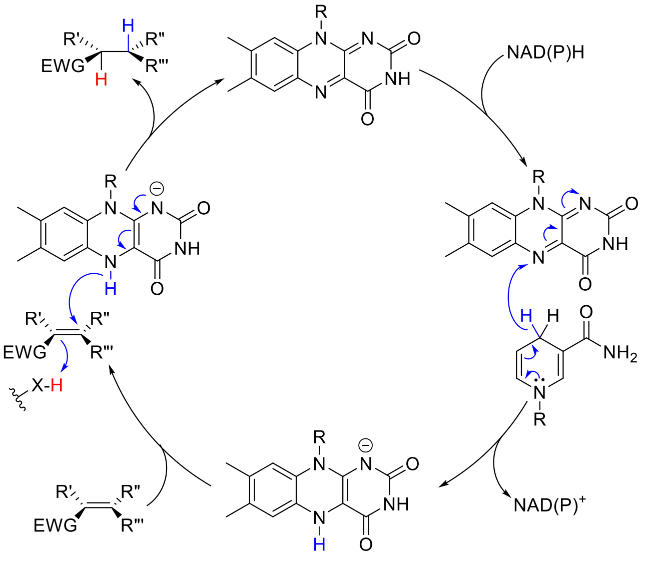

Figure S4. Representative mechanism of ER-catalysed *trans*-hydrogenation of conjugated C=C-double bonds. X = Tyr or H_2O.

2.3. Imine reductases

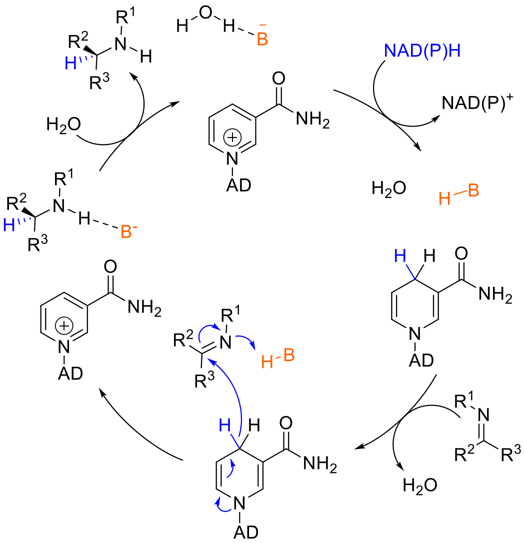

Figure S5. Representative simplified mechanism of IRED-catalysed imine reduction. B = Tyr, His, H_2O.

2.4. Transaminases

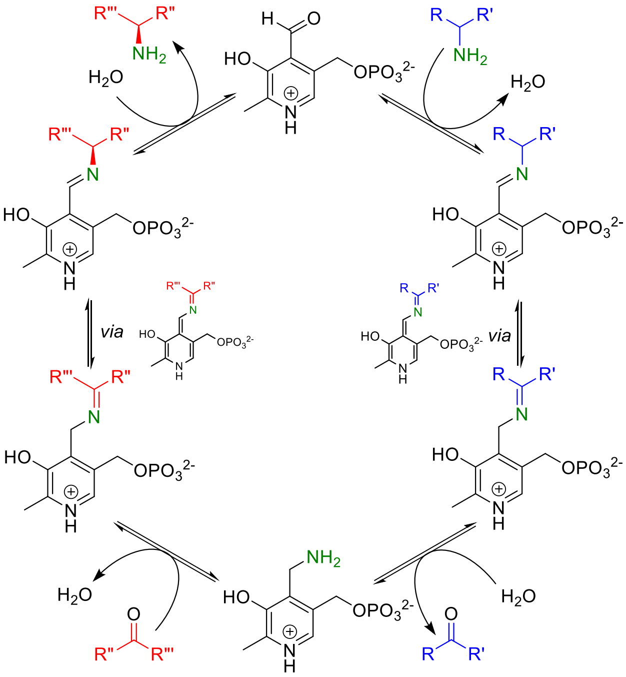

Figure S6. Simplified mechanism of transaminase-catalysed reductive amination of ketones.

2.5. Amine dehydrogenases

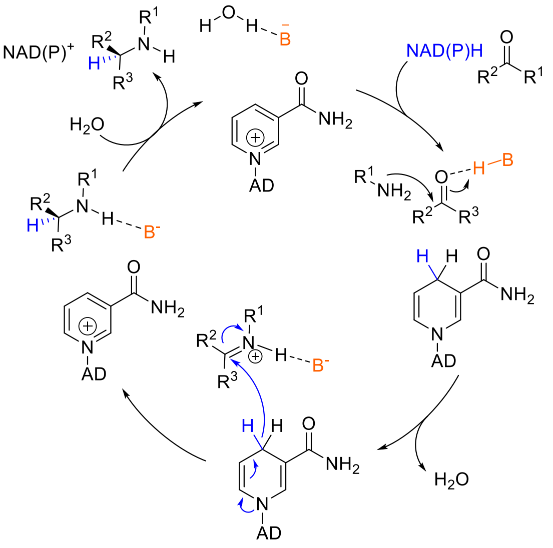

Figure S7. Simplified mechanism of AmDH-catalysed reductive amination of ketones. R^1 = H or alkyl group.

2.6. Carboxylic acid reductases

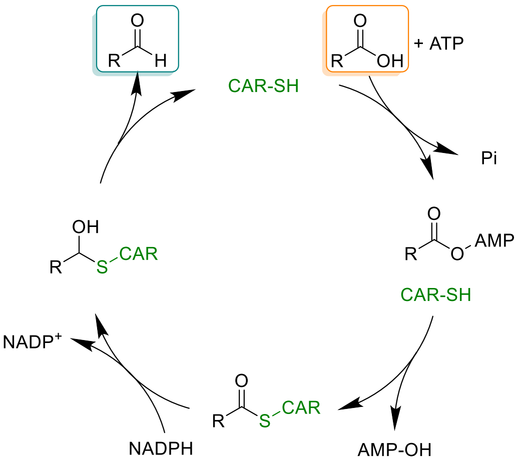

Figure S8. Simplified mechanism of the CAR-mediated activation and reduction of carboxylic acids.
